# Broadband acoustic invisibility and illusions

**DOI:** 10.1126/sciadv.abi9627

**Published:** 2021-09-10

**Authors:** Theodor S. Becker, Dirk-Jan van Manen, Thomas Haag, Christoph Bärlocher, Xun Li, Nele Börsing, Andrew Curtis, Marc Serra-Garcia, Johan O. A. Robertsson

**Affiliations:** 1 Institute of Geophysics, ETH Zürich, 8092 Zürich, Switzerland.; 2 Grant Institute of Geoscience, University of Edinburgh, Edinburgh EH9 3FE, UK.

## Abstract

Rendering objects invisible to impinging acoustic waves (cloaking) and creating acoustic illusions (holography) has been attempted using active and passive approaches. While most passive methods are inflexible and applicable only to narrow frequency bands, active approaches attempt to respond dynamically, interfering with broadband incident or scattered wavefields by emitting secondary waves. Without prior knowledge of the primary wavefield, the signals for the secondary sources need to be estimated and adapted in real time. This has thus far impeded active cloaking and holography for broadband wavefields. We present experimental results of active acoustic cloaking and holography without prior knowledge of the wavefield so that objects remain invisible and illusions intact even for broadband moving sources. This opens previously inaccessible research directions and facilitates practical applications including architectural acoustics, education, and stealth.

## Teaser

The active manipulation of sound waves replaces physical with virtual media to create acoustic invisibility and illusions.

## INTRODUCTION

**Fig. 1.**
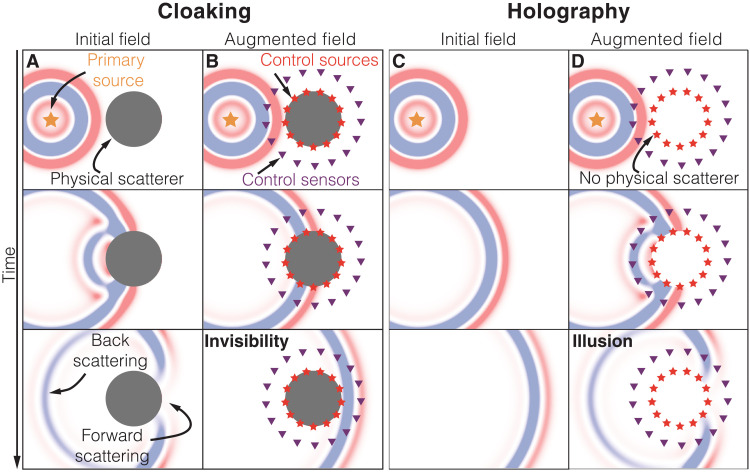
Simulations demonstrating active cloaking and holography. A primary source emits an initial wavefield, which is scattered by an object in the case of cloaking (**A**) or propagates unobstructed in the case of holography (**C**). Active control sources augment the incident wavefield to either cloak the scattering object (**B**) or create a hologram of an object that is not physically present (**D**). The input to the control sources is obtained by real-time forward extrapolation using measurements of the control sensors.

Rendering objects invisible to acoustic, elastic, or electromagnetic waves (cloaking) or making objects appear where there are none (holography) is of immediate scientific and technological interest. Sophisticated cloaks and holograms relying on real-time wavefield manipulation (Fig. 1) remained science fiction until theoretical advances, particularly in the field of transformation optics (*1*, *2*), enabled scientists to create the first physical invisibility and illusion devices (*3*–*9*). Existing devices rely on the control of wavefields with passive or active methods but have serious limitations. Passive methods (*1*, *5*–*7*, *9*–*15*) are based on tailored materials to manipulate impinging wavefields. While most of these approaches work efficiently only over narrow frequency bands and suffer from internal dissipation (*16*, *17*), some approaches, particularly those based on transformation acoustics and scattering cancellation (*8*, *15*, *18*), can be effective over a broader frequency range. However, passive methods require the meticulous design and construction of materials that manipulate the wavefield. Hence, they are inflexible and do not adapt to changing incident wavefields or requirements. Active methods (*3*, *4*, *19*–*24*) rely on the emission of a secondary wavefield that (destructively) interferes with the primary field. The secondary wavefield is typically created with control sources distributed on the boundary or across the area of interest (Fig. 1). Active methods provide a more flexible and dynamic control of wavefields and can, in principle, react to changes in the incident field in real time. However, existing active cloaking and holography devices have considerable limitations: They rely on a priori knowledge of the incident or scattered wavefields and hence fail if the characteristics of the primary energy sources change or are unknown (*23*–*25*), which is the case for many conceivable applications. In such a scenario, the signals for the control sources need to be estimated and updated in real time. To our knowledge, Friot *et al.* (*4*) are the only authors who have experimentally demonstrated active cloaking of acoustic waves without a priori knowledge of the incident wavefield beyond one dimension (1D). However, they used a control algorithm that relies on a nondeterministic optimization approach that reduces the scattered field in certain areas, while it actually increases the field in other locations. Moreover, in these experiments, the forward-scattered field is not controlled at all, the results are not spectrally broadband, and some knowledge of the signal emitted by the primary source is required. These assumptions compromise the cloak and limit its practical use.

**Fig. 2.**
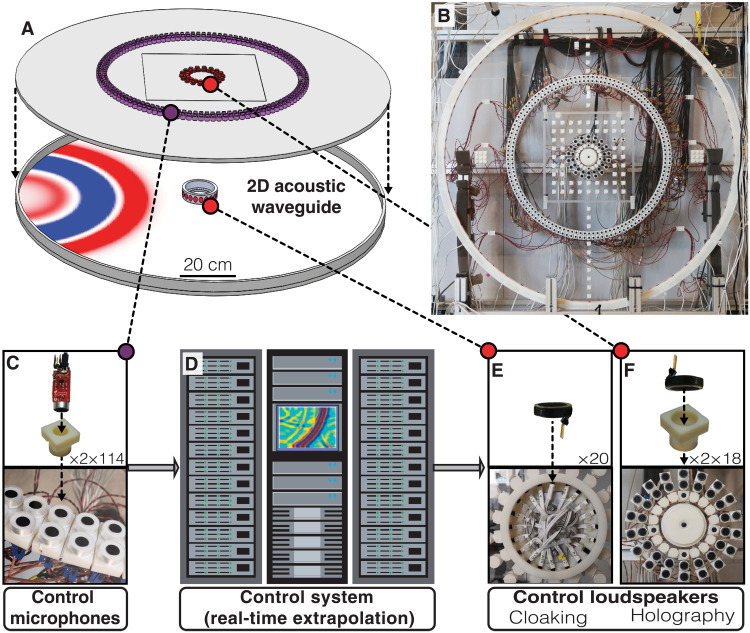
Experimental setup for active acoustic cloaking and holography. A schematic and photograph are shown in (**A**) and (**B**), respectively. Two circular arrays with each 114 microphones record the pressure fields (**C**). Using the recordings, an FPGA-based low-latency control system (**D**) performs the real-time wavefield extrapolation to predict the input signals for the control loudspeakers (**E** and **F**). Photo credit: Theodor S. Becker, ETH Zürich.

We consider a fundamentally different approach to acoustic cloaking and holography that relies on virtually replacing part of a physical medium by a desired, virtual medium. We achieve this by complementing the acoustic waves propagating in the physical medium with a real-time simulation of the waves propagating in the virtual medium. The simulation is performed on a massively parallelized, low-latency control system based on field-programmable gate arrays [FPGAs; see (*26*) for details]. An active, fully deterministic, and global control loop [(*27*–*29*) and Supplementary Materials] links the physical and virtual media in real time by extrapolating particle velocity and pressure wavefields from an acoustically transparent array of microphones to an array of control loudspeakers surrounding the region to be replaced (see Fig. 2 and the Supplementary Materials for details). Using this approach, we demonstrate in 2D acoustic experiments that a physical scattering object can be replaced with a virtual homogeneous background medium in real time, thereby hiding the object from broadband acoustic waves (cloaking). In a second set of experiments, we replace part of a physical homogeneous medium by a virtual scattering object, thereby creating an acoustic illusion of an object that is not physically present (holography). Because of the broadband nature of the control loop and in contrast to other cloaking approaches, this requires neither a priori knowledge of the primary energy source nor of the scattered wavefields, and the approach holds even for primary sources, whose locations change over time.

## RESULTS

**Fig. 3.**
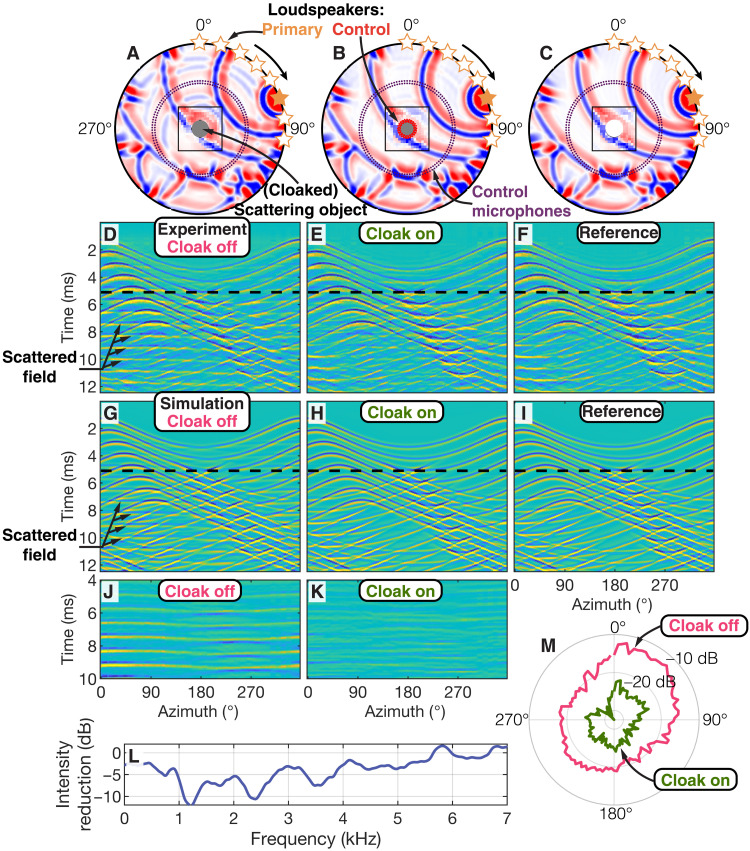
Cloaking of a rigid object. (**A** to **C**) Experimental setup and snapshots at 5.1 ms of the experimental data (black square) and corresponding simulations. Experimental and simulated pressure fields at the outer circular microphone array are shown in (**D** to **F**) and (**G** to **I**), respectively. (A, D, and G) The object is present inside the waveguide, and the cloak is off. (B, E, and H) The cloak is switched on; consequently, the object is hidden. (C, F, and I) References without object inside the waveguide. (**J** and **K**) Scattered fields without and with cloak, respectively. (**L**) Reduction in scattered acoustic intensity due to active cloaking as a function of frequency (see Eq. 1). (**M**) Acoustic intensity in (J) and (K) as a function of angle (Eqs. 2 and 3).

We first demonstrate cloaking of a quasi-rigid, circular scatterer with a diameter of 12.6 cm placed in the center of an air-filled, 2D acoustic waveguide. The boundary of the scatterer is lined with 20 secondary loudspeakers (Fig. 2E). A primary wavefield is emitted by eight individual loudspeakers located along the perimeter of the experiment across an arc of 96° (Fig. 3A). All loudspeakers emit the same broadband wavelet [described by the second derivative of a Gaussian function (*30*)] with a center frequency, *f*_c_, of 3 kHz but with an increasing time delay. This mimics a primary source that changes its location with an effective velocity of 173 m/s, sending a broadband pulse from each new location. First, the control loudspeakers surrounding the scatterer are inactive. Consequently, the rigid scatterer disturbs the incident wavefield, causing back scattering and forward scattering (Fig. 3, A, D, G, and J). In a second experiment, the primary loudspeakers emit the same primary wavefield, but this time, the control loudspeakers complement the scattered field based on the particle velocity field extrapolated in real time from two circular microphone arrays enclosing the scatterer (Fig. 3, B, E, H, and K). Since the Green’s functions used for the extrapolation simulate a homogeneous, air-filled medium, the scattering object is effectively replaced by air through the emission of the secondary wavefield and therefore rendered acoustically invisible for an observer outside of the control loudspeaker array. For comparison, we emit the same primary field but remove the scattering object and secondary loudspeakers from the waveguide (Fig. 3, C, F, and I). The resulting acoustic wavefield closely resembles the wavefield obtained with enabled secondary loudspeakers, confirming that the scatterer is rendered acoustically invisible. Active cloaking suppresses not only the scattered field arising from the direct wave propagating between the primary source and the scattering object but also that caused by the echoes from the boundary of the waveguide at later times (see fig. S1). The cloak is effective over a broad range of frequencies and all angles with a reduction in mean scattered acoustic intensity of −8.4 dB (Eq. 1 and Fig. 3, L and M). Note that the scatterer remains acoustically invisible despite the change of location of the primary source, demonstrating that our approach does not rely on any a priori knowledge of the incident or scattered fields. Finite-element simulations (see Materials and Methods) replicating the three experiments are in excellent agreement with the experimental results (Fig. 3, G to I).

**Fig. 4.**
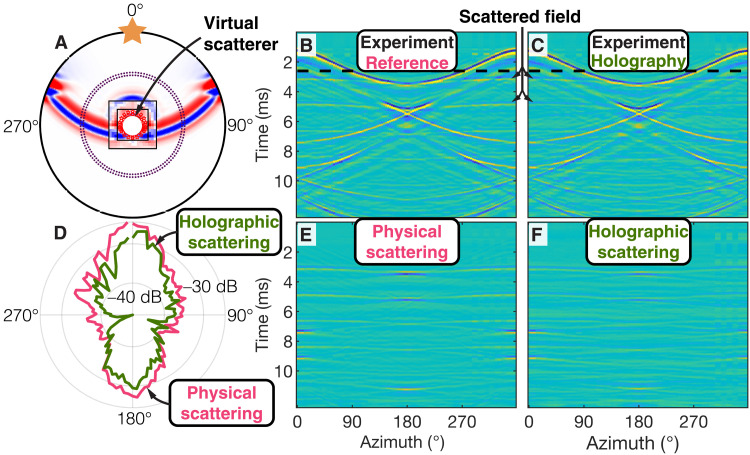
Creation of acoustic holograms at a sound-transparent surface. (**A**) Experimental setup and snapshots at 2.5 ms of the experimental data (black square) and corresponding simulations. Symbols follow those of Fig. 3. (**B**) Reference experiment with a physical object present inside waveguide. (**C**) Active creation of a hologram by enabling control loudspeakers (no physical object inside the waveguide). (**D**) Angular distribution of acoustic intensity (Eqs. 3 and 4) in the scattered fields due to a physical (**E**) and virtual (**F**) scatterer.

In a second suite of experiments, we demonstrate the ability to create broadband acoustic holograms. For these experiments, the Green’s functions used for the real-time wavefield extrapolation simulate wavefield interactions with virtual objects located within the control loudspeaker array. Since, in this case, no physical boundary exists at the location of the loudspeakers, wavefields are controlled at a sound-transparent emitting surface. This requires the dual emission (and extrapolation) of particle velocity and pressure as the signatures of collocated monopole and dipole sources [(*29*) and Supplementary Materials]. Consequently, in these experiments, the wavefield is controlled with two closely spaced circular loudspeaker arrays mounted flush with the planar boundary of the waveguide to create effective dipole and monopole sources (see Fig. 2F and Supplementary Materials). We choose extrapolation Green’s functions that represent a virtual rigid scatterer with the same geometry as the physical scatterer used in the cloaking experiments (i.e., circular, 12.6 cm in diameter, and rigid boundary; geometry shown in Fig. 4A). This time, a single stationary loudspeaker placed at [0, 0.66] m emits a primary wavefield with a wavelet with *f*_c_ = 3 kHz. The pressure field is measured by the microphone arrays and extrapolated to the sound-transparent emitting surface in real time, where the resulting virtually scattered field is emitted by the secondary loudspeakers. Consequently, a hologram of the virtual scatterer is created: The total wavefield now contains interactions of the primary loudspeaker with the virtual object. The resulting acoustic wavefield and angular distribution of virtually scattered intensity are in excellent agreement with measurements made with a physical scatterer inside the waveguide (Fig. 4, B to F), further underlining the successful creation of a broadband acoustic hologram.

## DISCUSSION

We demonstrated the ability to replace part of a physical medium with a virtual medium through real-time and broadband wavefield control. In these experiments, we either replaced a physical scatterer by virtual air, thereby rendering the scatterer acoustically invisible, or created the illusion of a virtual rigid object located within the array of control loudspeakers. The presented results demonstrate effectiveness over a broad frequency range of more than 3.5 octaves, with upper frequency limits of 8.7 kHz (cloaking) and 5.9 kHz (holography), consistent with classical sampling theory (see Supplementary Materials for details). However, modifying the experimental setup can relax these limits.

Hardware perturbations from the ideal case are, of course, always present. For instance, results will deteriorate with malfunctioning sources or receivers. However, loudspeakers and microphones are usually very reliable; we have not observed failures during our work. Moreover, regular tests (e.g., subsequently emitting each source, recording on all microphones, and comparing the results to a set of reference measurements) can help to identify and replace such failing components. Some applications, however, might not allow ad hoc hardware replacements and require operation with a reduced number of sources or receivers. In this case, a modification of the extrapolation Green’s functions can compensate for such drop-outs as long as the number of failing components is reasonable (e.g., every second loudspeaker failing, leading to 1.4 loudspeakers per wavelength), as was demonstrated in previous physical tests of the underlying control algorithm (*31*).

The proposed methodology opens up previously inaccessible research in wave physics, particularly because any desired virtual medium of arbitrary complexity, including nonlinear or nonphysical (e.g., energy-gaining) media, can be inserted into the physical medium within the loudspeaker arrays, as long as the medium can be described by an appropriate set of extrapolation Green’s functions. By physically recording the scattering Green’s functions of an existing object and using those Green’s functions for the real-time extrapolation in the presented holography approach, physical objects can be acoustically cloned and reproduced in a different environment (see Supplementary Materials for details). Moreover, the methodology is not restricted to experiments in air. For instance, it can also be used to create underwater acoustic cloaks and illusions. While the substantially increased speed of sound in water dictates a smaller latency for the real-time wavefield extrapolation, moving the control receivers further away from the control sources or using hardware with flatter frequency responses (i.e., requiring shorter filters and thus less latency for their correction) counteracts these additional challenges. In principle, the methodology also extends to elastodynamic and electromagnetic waves, but a practical realization is considerably more challenging. The approach has immediate practical implications, including hiding arbitrary objects from interrogating devices, improving architectural acoustics, creating invisible sensors, and creating holograms for communication, educational, entertainment, and stealth purposes.

## MATERIALS AND METHODS

### Experimental design

We use an air-filled acoustic waveguide composed of two parallel polymethyl methacrylate (PMMA) plates with a spacing of *a* = 2.5 cm for the presented cloaking and holography experiments. See Fig. 2 for photographs of the experimental setup and hardware components. The boundary of the experimental domain consists of a 3D printed ring with a radius of 66.2 cm made of the synthetic resin VeroWhite. The propagation of waves between the two PMMA plates can be considered 2D as long as the signals below the cutoff frequency of the fundamental mode are used, which is given by *f*_c_ = 0.5*ca*^−1^ (*32*), where *c* is the speed of sound. For our experiments this results in *f*_c_ ≈ 6.9 kHz. Instead of directly measuring the normal particle velocity and pressure on *S*^rec^, as dictated by the theory of the underlying control algorithm (see Supplementary Materials for details), we use two circular arrays of pressure microphones with radii of 35.3 and 37.3 cm, respectively, each consisting of 114 electret condenser microphones (SparkFun Electronics, BOB-12758 and COM-08635; Fig. 2C). The normal particle velocity is then approximated using the pressure gradient in the normal direction (*26*, *33*). All microphones are mounted flush with the inside of the front PMMA plate to minimize their impact on the pressure field. In the case of cloaking experiments, the rigid emitting boundary at *S*^emt^ consists of a 3D printed ring with a radius of 6.4 cm, housing 20 loudspeakers (PUI Audio, AS01508MR-R; Fig. 2E). For the holography experiments, the transparent emitting boundary at *S*^emt^ consists of two separate circular source arrays with radii of 7.4 and 9.4 cm, respectively, each consisting of 18 loudspeakers mounted flush with the front PMMA plate (Fig. 2F). We produced all holes in the PMMA plate with a computerized numerical control (CNC) milling machine to ensure high accuracy and precision. All microphones and the loudspeakers of the holography experiment are mounted into custom-made, 3D printed supports (Fig. 2, C and F). The supports are wrapped with Teflon tape and then inserted into the holes in the PMMA plate. This ensures an accurate fit, closes holes, and reduces wavefield scattering. We validated the assumption of 2D wave propagation in the waveguide and the negligibility of scattering at the control hardware in previous experiments (*31*). The distance between *S*^rec^ and *S*^emt^ of approximately 30 cm allows a maximum total latency of about 870 μs (for experiments in air). Of these, the wavefield extrapolation consumes 200 μs, while the remaining 670 μs are available for real-time hardware corrections and estimation of normal particle velocity from pressure recordings. A massively parallelized, low-latency data acquisition and control system enabled by more than 500 National Instruments FlexRIO FPGAs runs the nonlocal control algorithm. The system supports simultaneous recording of 800 analog input channels and simultaneous emission of 800 analog output channels while operating at a 20-kHz sample rate [more details of the system architecture can be found in (*26*)]. The experiments and data acquisition are performed with LabVIEW.

### Numerical simulations

The numerical reference simulations in Figs. 3 and 4 and the computation of all extrapolation Green’s functions are performed with the finite-element simulation software COMSOL Multiphysics (version 5.5, Acoustics Module). The simulation domain consists of air with a density of 1.225 kg/m^3^ and a speed of sound of 347 m/s. The circular scatterer is simulated as a sound-hard (i.e., rigid) object. The Green’s functions are obtained by simulating impulsive monopole and dipole sources at the location of the control loudspeakers, recording pressure and particle velocity at the location of the control microphones, and applying source-receiver reciprocity.

### Scattered acoustic intensity and repeatability

We assess the effectiveness of the broadband active cloak in terms of the reduction in scattered acoustic intensity, *I*_R_, given by

$$I_{R}=\frac{1}{N_{r}}\sum_{j=1}^{N_{r}}[I_{\text{scat},\text{cloak}}(\theta_{j})-I_{\text{scat},\text{phys}}(\theta_{j})]$$ (1)

where *I*_scat,cloak_(θ*_j_*) and *I*_scat,phys_(θ*_j_*) are the scattered acoustic intensities in the cloaked and scattering case, respectively, which are given by

$$I_{\text{scat},\text{cloak}}(\theta_{j})=10{\text{log}}_{10}\Sigma_{i=1}^{N_{t}}\;{\hat{p}}_{\text{scat},\text{cloak}}^{2}(t_{i},\theta_{j})$$ (2)

and

$$I_{\text{scat},\text{phys}}(\theta_{j})=10{\text{log}}_{10}\Sigma_{i=1}^{N_{t}}\;{\hat{p}}_{\text{scat},\text{phys}}^{2}(t_{i},\theta_{j})$$ (3)

Here, ${\hat{p}}_{\text{scat},\text{cloak}}$ and ${\hat{p}}_{\text{scat},\text{phys}}$ are the discrete (residual) scattered pressure fields due to the presence of the physical scatterer with and without active cloak, respectively. The quantities ${\hat{p}}_{\text{scat},\text{cloak}}$ and ${\hat{p}}_{\text{scat},\text{phys}}$ are obtained according to ${\hat{p}}_{\text{scat},\text{cloak}}={\hat{p}}_{\text{cloak}}-{\hat{p}}_{\text{hom}}$ and ${\hat{p}}_{\text{scat},\text{phys}}={\hat{p}}_{\text{phys}}-{\hat{p}}_{\text{hom}}$, i.e., by subtracting from the total pressure fields the pressure field, ${\hat{p}}_{\text{hom}}$, measured in a reference experiment without physical scatterer present (see Fig. 3, J and K). In Eqs. 1 to 3, θ*_j_* denotes the azimuth of the *j*th microphone on the outer circular microphone array with a total of *N*_r_ = 114 pressure field microphones (see Fig. 2), *t_i_* are sample times, and *N*_t_ is the total number of time samples per recording. To discard periods without signal in the calculation, the recordings are first filtered with a bandpass filter from 0.3 to 10 kHz and the time samples limited to those for which $|{\hat{p}}_{\text{scat},\text{phys}}|>0.1\;\text{max}\;|{\hat{p}}_{\text{scat},\text{phys}}|$. A similar expression for reduction in scattered field intensity as a function of frequency can be derived by first transforming ${\hat{p}}_{\text{scat},\text{cloak}}$ and ${\hat{p}}_{\text{scat},\text{phys}}$ to the frequency domain and omitting the summation over time samples (see Fig. 3L).

**Fig. 5.**
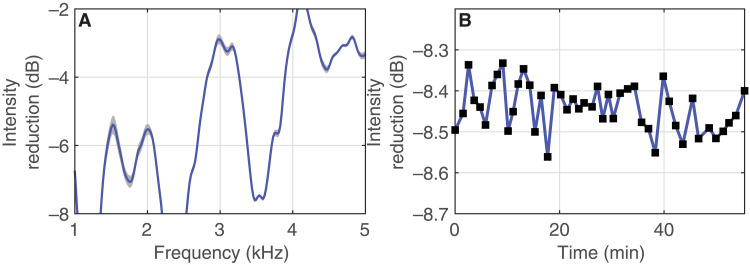
Error analysis for active broadband cloaking experiments. (**A**) Mean intensity reduction of the scattered field with enabled cloak for 46 realizations of the same cloaking experiment (blue line) and the according SD (gray area). (**B**) Intensity reduction of the same 46 realizations over time.

We repeat the experiments with and without active cloak a total of 46 times over the duration of approximately 1 hour to analyze repeatability. This leads to a mean reduction in scattered acoustic intensity of −8.4 dB with an SD of 0.05 dB. These findings highlight the strong reduction in scattered field when the cloak is active while maintaining a high repeatability across experiments (see Fig. 5).

To assess the quality of the holography results, the scattered acoustic intensity due to holographic scattering is calculated according to

$$I_{\text{scat},\text{holo}}(\theta_{j})=10{\text{log}}_{10}\Sigma_{i=1}^{N_{t}}\;{\hat{p}}_{\text{scat},\text{holo}}^{2}(t_{i},\theta_{j})$$ (4)

with ${\hat{p}}_{\text{scat},\text{holo}}={\hat{p}}_{\text{holo}}-{\hat{p}}_{\text{hom}}$ (Fig. 4F). Here, ${\hat{p}}_{\text{scat},\text{holo}}$ is the pressure field due to holographic scattering in the absence of a physical scatterer, and ${\hat{p}}_{\text{holo}}$ is the total pressure field in the holography experiment. The acoustic intensities due to holographic (Eq. 4) and physical (Eq. 3) scattering are compared in Fig. 4D and were found to be in good agreement.

## Supplementary Materials

### This PDF file includes:

The control algorithm: Immersive boundary conditions Physical implementation of immersive boundary conditions Frequency limitations of immersive experiments Figs. S1 to S3 References
