# Supplementary material for: Broadband acoustic invisibility and illusions
Source: Sci Adv. 2021 Sep 10;7(37):eabi9627. doi: 10.1126/sciadv.abi9627 (PMC8442923; doi:10.1126/sciadv.abi9627)
Supplement: Supplementary file 1 — The control algorithm: Immersive boundary conditions Physical implementation of immersive boundary conditions Frequency limitations of immersive experiments Figs. S1 to S3 References [file sciadv.abi9627_sm.pdf]

Science Advances
AAAS

Supplementary Materials for

Broadband acoustic invisibility and illusions

Theodor S. Becker*, Dirk-Jan van Manen, Thomas Haag, Christoph Bärlocher, Xun Li, Nele Börsing, Andrew Curtis, Marc Serra-Garcia, Johan O. A. Robertsson

*Corresponding author. Email: becker.theodor@gmail.com

Published 10 September 2021, *Sci. Adv.* **7**, eabi9627 (2021)
DOI: 10.1126/sciadv.abi9627

This PDF file includes:

The control algorithm: Immersive boundary conditions
Physical implementation of immersive boundary conditions
Frequency limitations of immersive experiments
Figs. S1 to S3
References

The control algorithm: Immersive boundary conditions

The control algorithm underlying the experiments of this study is based on immersive boundary conditions (IBCs). IBCs were initially thought-out to obtain non-reflecting domain boundaries in numerical simulations and to embed truncated modeling domains in larger surrounding background media (*28*). This allows broadband waves from unknown sources to propagate seamlessly between the truncated domain and the background medium, including arbitrary-order scattering interactions due to the recursive nature of the boundary conditions. Ref. (*34*) realized that such boundary conditions could also serve as the control algorithm to embed physical wave propagation experiments in surrounding numerical simulations, thereby removing adverse boundary reflections from the experimental domain, overcoming certain wavelength limitations of traditional laboratory experiments, and fully capturing all wave interactions with the numerical background medium. Subsequently, this was demonstrated in 1D and 2D acoustic (*26, 33*) and 1D elastic (*35*) experiments by embedding physical waveguides in surrounding virtual media. Moreover, Ref. (*29*) demonstrated in numerical simulations that IBCs can also be employed for cloaking and holography of broadband wavefields, which was experimentally verified in 1D acoustic experiments by (*36*), for which the underlying wavefield extrapolation reduces to a trivial convolution. In the following, we first summarize the use of IBCs for cloaking of rigid objects and then for creating holograms at a sound-transparent boundary. More detailed derivations can be found in previous studies (*28, 29*).

Cloaking of a rigid object

Consider an object embedded in a homogeneous background medium that scatters an incident wavefield (Fig. S2A). The pressure field of this *initial* scattering state is denoted with p_{ini}. By emitting appropriate boundary conditions on the surface S^{emt} enclosing the object, the imprint of the scattering object can be removed from p_{ini}, thereby hiding the object from an observer outside S^{emt} (Fig. S2, B and C). The pressure of this *augmented* state in which the object is cloaked is denoted with p_{aug} and can be understood as the superposition of the pressure in the scattering state and the pressure associated with the wavefield radiated by the boundary conditions, p_{ibc}:

$$p_{\text{aug}} = p_{\text{ini}} + p_{\text{ibc}}. \tag{5}$$

Hence, the goal of IBCs as the control algorithm is to inject the correct boundary wavefield p_{ibc} on S^{emt} to obtain the desired wavefield p_{aug}. The boundary wavefield can be found by differencing the pressure field representations for the initial and the augmented state (*29*). If the surface S^{emt} aligns with the rigid boundary of the scattering object (i.e., the normal particle velocity on the boundary vanishes), the boundary pressure field is given by (*29*):

$$p_{\text{ibc,rigid}}(\boldsymbol{x}', t) = \oint_{S^{\text{emt}}} [G^q_{\text{ini}}(\boldsymbol{x}', \boldsymbol{x}, t) * v_{\text{aug},i}(\boldsymbol{x}, t)] n_i \, dS, \tag{6}$$

where $v_{\text{aug},i}(\mathbf{x}, t)$ is the i-th component of the particle velocity in the augmented (i.e., homogeneous) medium, $G^q_{\text{ini}}(\mathbf{x}', \mathbf{x}, t)$ is the acoustic pressure impulse response (Green's functions) of the medium at $\mathbf{x}'$ due to a point source of volume injection rate density, n_i denotes the i-th

component of the outward-directed normal on S^{emt}, and an asterisk represents temporal convolution. Equation (6) can be interpreted as dense distributions of secondary monopole sources (G^q_{ini}) on S^{emt} with source strength $v_{\mathrm{aug},i}(\mathbf{x},t)$. Despite the apparent simplicity of this equation, its implementation in physical real-time experiments poses a significant challenge as the normal particle velocity on S^{emt} needs to be known prior to the arrival of the respective waves. To that end, we introduce a sound-transparent auxiliary recording surface S^{rec} enclosing S^{emt} (Fig. S2), from which normal particle velocity and pressure wavefields are extrapolated to S^{emt} using a Kirchhoff-Helmholtz extrapolation integral (*29*):

$$\begin{aligned} v_{\mathrm{aug},i}(\boldsymbol{x}^{\mathrm{emt}},t) \\ &= \oint_{S^{\mathrm{rec}}} [\Gamma^q_{\mathrm{aug},i}(\boldsymbol{x}^{\mathrm{emt}},\boldsymbol{x},t) * v_{\mathrm{aug},m}(\boldsymbol{x},t) \\ &\qquad + \Gamma^f_{\mathrm{aug},i,m}(\boldsymbol{x}^{\mathrm{emt}},\boldsymbol{x},t) * p_{\mathrm{aug}}(\boldsymbol{x},t)] n_m \, dS. \end{aligned} \tag{7}$$

Here, $\Gamma^q_{\mathrm{aug},i}$ and $\Gamma^f_{\mathrm{aug},i,m}$ represent the i-th component of the particle velocity impulse response (Green's functions) due to a monopole source and an m-directed point-force (dipole) source, respectively, $v_{\mathrm{aug},m}(\boldsymbol{x},t)$ and $p_{\mathrm{aug}}(\boldsymbol{x},t)$ are the normal particle velocity and pressure of the augmented state at S^{rec}, and n_m is the m-th component of the outward-pointing normal on S^{rec}. The Green's functions in Eq. (7) represent the desired medium inside S^{emt}, which is a homogeneous volume in the case of cloaking, but in principle, the Green's functions can represent arbitrarily complex, even nonphysical media within S^{emt}. This leads to an intentional misrepresentation of the actual medium inside S^{emt}. It is worth noting that the simultaneous extrapolation of particle velocity and pressure implicitly separates the wavefield into ingoing and outgoing components at S^{rec} and only the ingoing component is extrapolated to the secondary sources at S^{emt}, while the outgoing components sum to *zero* (*26*).

Holography at a sound-transparent surface

In some sense, the creation of holograms is the opposite to cloaking: while the initial state is homogeneous, the augmented state contains a desired scattering object (Fig. S2, D-F). The boundary wavefield required to create acoustic holograms can also be found by differencing the pressure field representations in the initial and the augmented states. This time, we do not impose any boundary conditions on S^{emt}, because in order to create holograms of virtual objects that are not physically present, the emitting surface is, by definition, a transparent surface (Fig. S2E). In that case the required boundary wavefield is given by (*29*):

$$p_{\mathrm{ibc,transparent}}(\boldsymbol{x'},t) = \oint_{S^{\mathrm{emt}}} [G^q_{\mathrm{ini}}(\boldsymbol{x}',\boldsymbol{x},t) * v_i(\boldsymbol{x},t) + G^{f,i}_{\mathrm{ini}}(\boldsymbol{x}',\boldsymbol{x},t) * p(\boldsymbol{x},t)] n_i \, dS, \tag{8}$$

with $v_i(\boldsymbol{x},t) = v_{\mathrm{aug},i}(\boldsymbol{x},t) - v_{\mathrm{ini},i}(\boldsymbol{x},t)$ and $p(\boldsymbol{x},t) = p_{\mathrm{aug}}(\boldsymbol{x},t) - p_{\mathrm{ini}}(\boldsymbol{x},t)$, where $v_{\mathrm{aug},i}(\mathbf{x},t)$, $v_{\mathrm{ini},i}(\mathbf{x},t)$, $p_{\mathrm{aug}}(\boldsymbol{x},t)$ and $p_{\mathrm{ini}}(\boldsymbol{x},t)$ are the i-th component of the particle velocity and the pressure in the augmented and initial state, respectively, and $G^{f,i}_{\mathrm{ini}}(\boldsymbol{x}',\boldsymbol{x},t)$ is the acoustic pressure impulse response at $\mathbf{x}'$ due to an i-directed force at $\mathbf{x}$. Note that, while Eq. (6) only requires

monopole sources at the rigid boundary S^{emt}, the control of waves at a sound-transparent boundary requires monopole and dipole sources with source signatures $v_i(\boldsymbol{x},t)$ and $p(\boldsymbol{x},t)$, respectively (Fig. S2E). For that reason, two circular source arrays are used for the holography experiments presented in this study in order to create effective dipole and monopole sources, while for the cloaking experiments a single array suffices. Moreover, for a transparent boundary, the difference of the wavefields in the augmented and initial state are required as the source strengths of the secon dary sources on S^{emt}. As a consequence, four extrapolation integrals (compared to one for a rigid boundary) need to be evaluated to predict the required wavefields on S^{emt}. However, the number of extrapolation integrals reduces to two by differencing the extrapolation Green's functions in the augmented and initial state instead of the extrapolated wavefields:

$$\begin{aligned} &v_i(\boldsymbol{x}^{\text{emt}},t) \\ &\quad= \oint_{S^{\text{rec}}} [\bar{\Gamma}_i^q(\boldsymbol{x}^{\text{emt}},\boldsymbol{x},t) * v_{\text{aug},m}(\boldsymbol{x},t) \\ &\qquad\qquad + \bar{\Gamma}_{i,m}^f(\boldsymbol{x}^{\text{emt}},\boldsymbol{x},t) * p_{\text{aug}}(\boldsymbol{x},t)] n_m \, dS. \end{aligned} \tag{9}$$

and

$$\begin{aligned} &p(\boldsymbol{x}^{\text{emt}},t) \\ &\quad= \oint_{S^{\text{rec}}} [\bar{G}^q(\boldsymbol{x}^{\text{emt}},\boldsymbol{x},t) * v_{\text{aug},m}(\boldsymbol{x},t) \\ &\qquad\qquad + \bar{G}_m^f(\boldsymbol{x}^{\text{emt}},\boldsymbol{x},t) * p_{\text{aug}}(\boldsymbol{x},t)] n_m \, dS, \end{aligned} \tag{10}$$

where, $\bar{\Gamma}_i^q$, $\bar{\Gamma}_{i,m}^f$, $\bar{G}^q$ and $\bar{G}_m^f$ represent the i-th component of the *differenced* particle velocity and pressure impulse responses (Green's functions) due to a monopole source and an m-directed dipole source, respectively, with $\bar{\Gamma} = \Gamma_{\text{aug}} - \Gamma_{\text{ini}}$ and $\bar{G} = G_{\text{aug}} - G_{\text{ini}}$. This essentially isolates (and extrapolates) the contributions of the virtual scatterer, which are then superimposed on the primary wavefield to create the illusion of a scattering object, but does not extrapolate the direct waves between S^{rec} and S^{emt}.

According to Eqs. (7), (9), and (10), suitable sets of Green's functions for the extrapolation of particle velocity and pressure from the recording to the emitting surfaces are required to replace the medium within S^{emt} with a desired virtual medium. These Green's functions can be analytical, numerically simulated or physically measured [for instance using the approach outlined in (*37*)]. Here, we obtain the Green's functions by acoustic finite-element modeling of impulsive monopole and dipole sources on S^{emt}, recording pressure and particle velocity on S^{rec}, and applying source-receiver reciprocity. The numerical modeling is performed with COMSOL Multiphysics® (See Materials and Methods). If, instead, physically recorded scattering Green's functions of an object are used in Eqs. (9) and (10), the outlined holography approach would allow to acoustically reproduce the physical object in different physical or virtual environments, thereby acoustically cloning the object.

Physical implementation of immersive boundary conditions

The implementation of immersive boundary conditions in a physical wave propagation laboratory requires overcoming significant practical challenges. As seen above, the non-local nature of IBCs requires the evaluation of surface-integrals on a mathematically-closed and continuous recording surface in real-time so that control sources on the emitting surface can react appropriately to incoming waves. In our experiments, dense microphone and loudspeaker arrays replace these continuous surfaces. More concretely, measurements from 228 microphones are used for the real-time prediction of the required signals for 20 (cloaking) or 36 (holography) secondary loudspeakers. This constitutes a significant computational effort while requiring an extremely low latency. Hence, a custom-built, massively parallelized data acquisition and control system is used, on which Eqs. (7), (9), and (10) are implemented by discretizing them (in time and space) and replacing the surface integrals by matrix-vector multiplications. The extrapolation can then be performed recursively at each time step of an experiment (*26*).

To comply with the theory of IBCs, the physical experiments also require a range of hardware corrections, because "perfect" measurements of pressure and particle velocity, monopolar/dipolar sources, and "perfectly rigid boundaries" are idealized, theoretical constructs that are difficult to achieve in physical experiments. These corrections include the removal of microphone and loudspeaker transfer functions, particle velocity estimation from two parallel microphone arrays, and the creation of effective dipole sources using two parallel loudspeaker arrays. These corrections are represented by scalar operations or short, frequency-dependent filters, a few coefficients in length. The filters are obtained by matching observed output signals to desired input signals for multiple broadband wavelets in a least-squares sense. This circumvents having to know the transfer functions of the involved hardware *a priori* and implicitly removes them in a fully data-driven way (*31*). Due to the associative property of the convolution in Eqs. (7), (9), and (10), these corrections can be applied to the extrapolation Green's functions prior to an experiment and are then accounted for during the real-time extrapolation of the wavefield. Consequently, the corrections do not require additional computations or filtering operations at run-time. We also attempted to correct for the non-monopolar directivity pattern of the control loudspeakers by spatially filtering the Green's functions according to Ref. (*38*). However, we did not observe a significant decrease in residual errors and hence relied on frequency-dependent corrections only. The flowchart in Fig. S3 summarizes the hardware corrections and manipulations of the extrapolation Green's functions. Further details can be found in Refs. (*26, 31, 38*). The algorithm to process the extrapolation Green's functions and all involved filters are available in the file archive referenced in the acknowledgments.

Frequency limitations of immersive experiments

While the presented invisibility and illusions are effective over a wide frequency band (see e.g., Fig. 3L) and the underlying control algorithm is truly broadband from a theoretical point of view, classical sampling theory does impose limitations on the upper frequencies that can be effectively controlled with the proposed approach. The Nyquist-Shannon sampling theorem (*39*) dictates that a minimum of two control microphones and loudspeakers are required per minimum wavelength. In our experiments, control microphones are spaced 2.0 cm apart in the tangential direction. Control loudspeakers are spaced 2.0 cm apart for the cloaking experiments and approximately 2.9 cm apart for the holography experiments (see Materials and Methods). With a sound speed of $c = 347$ m s^{-1}, this corresponds to upper frequencies of approximately 8.7 kHz (cloaking) and 5.9 kHz (holography). These values are well below the frequency limitation imposed by the control system, which operates at a sampling frequency of 20 kHz (i.e., a Nyquist frequency of 10 kHz). Another, albeit loose, restriction on the upper frequency limit of the experiments is imposed by the separation distance between the two parallel plates that form the two-dimensional waveguide: the propagation of waves between the two plates can only be assumed two-dimensional for frequencies up to approximately 6.9 kHz (see Materials and Methods). To employ higher frequencies, the plates can either be moved closer together or higher-order modes need to be considered in the extrapolation Green's functions. Finally, the transfer functions of the employed control hardware, particularly those of the employed loudspeakers, constitute a lower limit for the usable frequency range of about 500 Hz. Below this, the loudspeakers are incapable of emitting significant acoustic energy. Note that the mentioned frequency limitations are not inherent to the presented approach itself, but are rather limits imposed by the current experimental setup. They can be relaxed, and the employable frequency range extended, by altering the control hardware and/or spatial discretization.

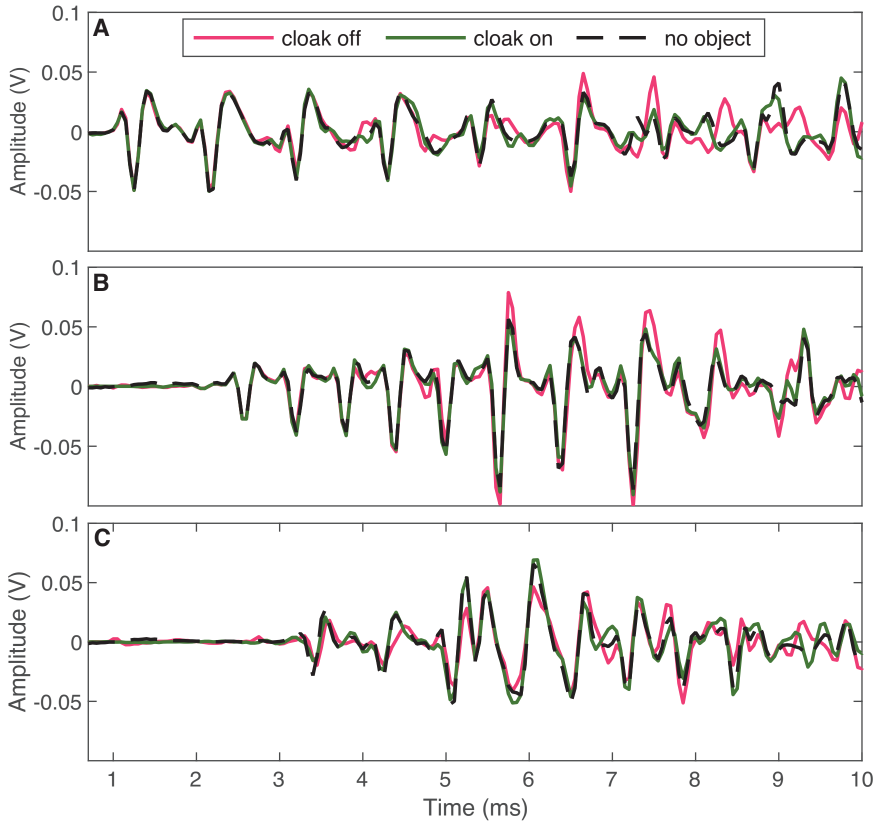

Figure S1: Time series measured with microphones on the outer circular array at azimuths of approximately 0° (A), 90° (B) and 180° (C). Note that the green and black dashed lines largely overlay due to the good agreement between augmented and reference results.

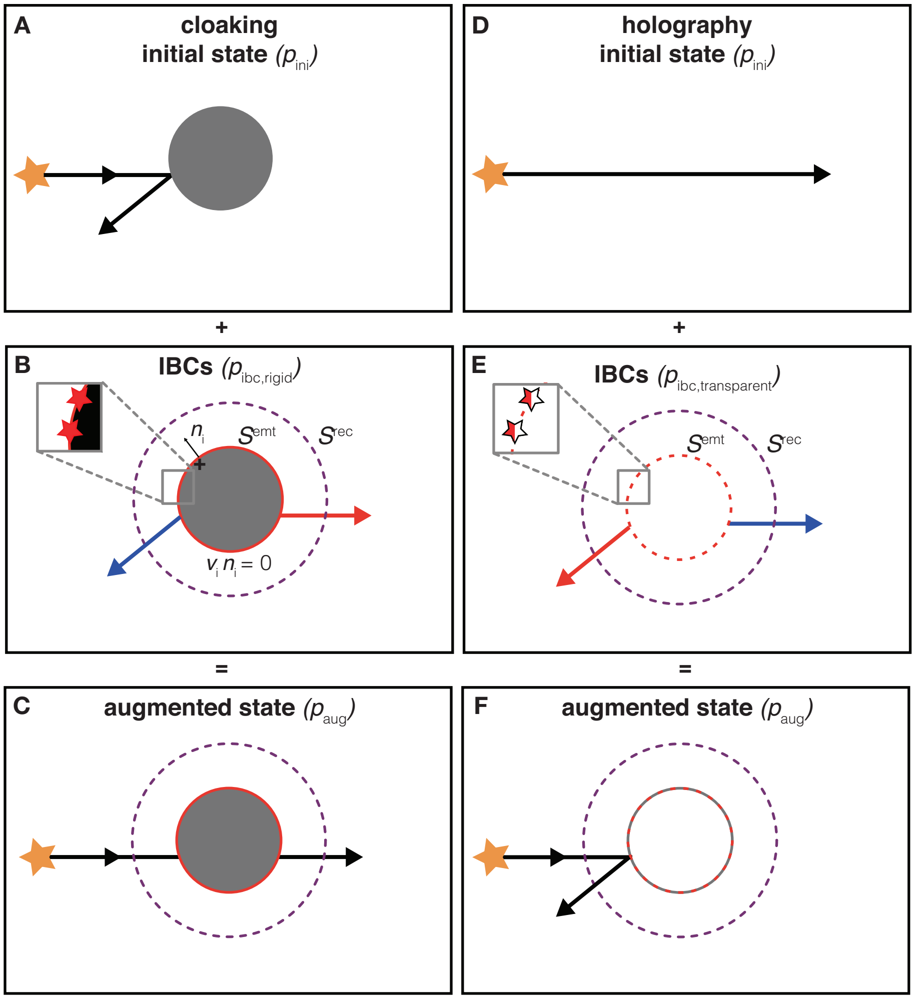

Figure S2: Illustration of wavefields for cloaking (A-C) and holography (D-F). (A): A rigid object (black circle) scatters the wavefield of a primary source (orange star) yielding the initial wavefield p_{ini}. (B): Monopole sources on the rigid boundary S^{emt} (red solid line / red stars) emit the boundary wavefield, $p_{\text{ibc,rigid}}$. This boundary wavefield interacts with the primary field, canceling the reflected waves (blue arrow) and reproducing transmitted waves (red arrow). The signatures of the monopole sources are obtained by forward extrapolation from the sound-transparent recording boundary S^{rec} (dashed purple line). (C): The superposition of wavefields p_{ini} and $p_{\text{ibc,rigid}}$ yields the augmented state, p_{aug}, for which the rigid scatterer is rendered invisible. For holography, an initially undisturbed wavefield propagating in a homogeneous medium (D) is augmented by the emission of collocated monopole and dipole sources on the (now) sound-transparent surface S^{emt} (E, red dashed line / white-red stars) to produce the forward-scattered (blue arrow) and back-scattered (red arrow) fields of a virtual object (E and F).

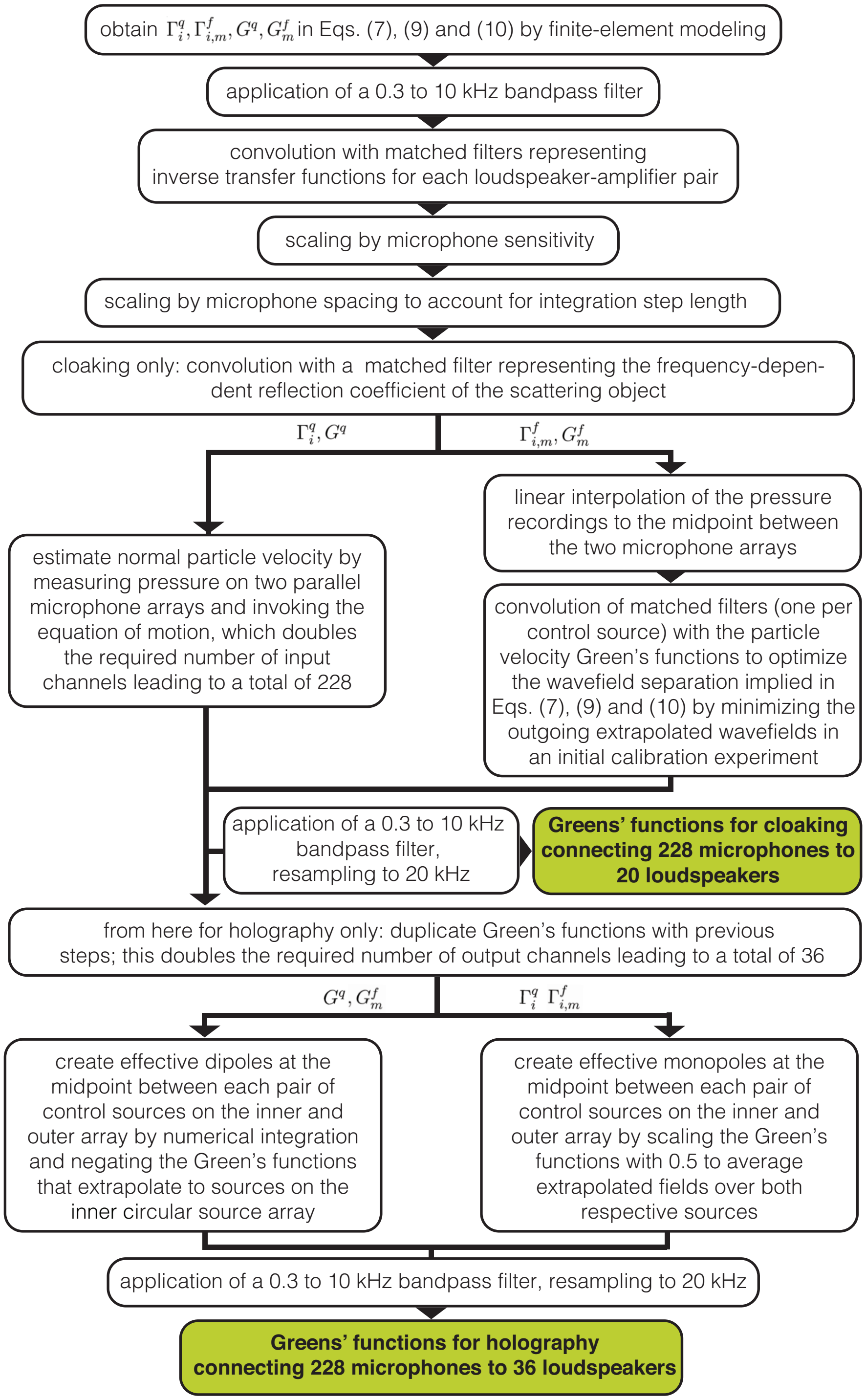

Figure S3: Flowchart describing the generation and processing of the extrapolation Green's functions for cloaking and holography experiments. For holography experiments, the workflow is repeated twice: once for Green's functions of the augmented state and once for Green's functions of the initial state.

REFERENCES AND NOTES

1. J. B. Pendry, D. Schurig, D. R. Smith, Controlling electromagnetic fields. *Science* **312**, 1780–1782 (2006).

2. U. Leonhardt, Optical conformal mapping. *Science* **312**, 1777–1780 (2006).

3. E. Friot, C. Bordier, Real-time active suppression of scattered acoustic radiation. *J. Sound Vib.* **278**, 563–580 (2004).

4. E. Friot, R. Guillermin, M. Winninger, Active control of scattered acoustic radiation: A real-time implementation for a three-dimensional object. *Acta Acust. United Ac.* **92**, 278–288 (2006).

5. D. Schurig, J. J. Mock, B. J. Justice, S. A. Cummer, J. B. Pendry, A. F. Starr, D. R. Smith, Metamaterial electromagnetic cloak at microwave frequencies. *Science* **314**, 977–980 (2006).

6. T. Ergin, N. Stenger, P. Brenner, J. B. Pendry, M. Wegener, Three-dimensional invisibility cloak at optical wavelengths. *Science* **328**, 337–339 (2010).

7. X. Chen, Y. Luo, J. Zhang, K. Jiang, J. B. Pendry, S. Zhang, Macroscopic invisibility cloaking of visible light. *Nat. Commun.* **2**, 176 (2011).

8. B.-I. Popa, L. Zigoneanu, S. A. Cummer, Experimental acoustic ground cloak in air. *Phys. Rev. Lett.* **106**, 253901 (2011).

9. H. Chen, B. Zheng, L. Shen, H. Wang, X. Zhang, N. I. Zheludev, B. Zhang, N. I. Zheludev, B. Zhang, Ray-optics cloaking devices for large objects in incoherent natural light. *Nat. Commun.* **4**, 2652 (2013).

10. W. X. Jiang, T. J. Cui, X. M. Yang, H. F. Ma, Q. Cheng, Shrinking an arbitrary object as one desires using metamaterials. *Appl. Phys. Lett.* **98**, 204101 (2011).

11. L. Sanchis, V. M. García-Chocano, R. Llopis-Pontiveros, A. Climente, J. Martínez-Pastor, F. Cervera, J. Sánchez-Dehesa, Three-dimensional axisymmetric cloak based on the cancellation of acoustic scattering from a sphere. *Phys. Rev. Lett.* **110**, 124301 (2013).

12. W. Kan, B. Liang, X. Zhu, R. Li, X. Zou, H. Wu, J. Yang, J. Cheng, Acoustic illusion near boundaries of arbitrary curved geometry. *Sci. Rep.* **3**, 1427 (2013).

13. X. Ni, Z. J. Wong, M. Mrejen, Y. Wang, X. Zhang, An ultrathin invisibility skin cloak for visible light. *Science* **349**, 1310–1314 (2015).

14. M. Kadic, R. Schittny, T. Bückmann, M. Wegener, Transformation wave physics. *Phil. Trans. R. Soc. A* **2016**, 335–368 (2016).

15. W. Kan, B. Liang, R. Li, X. Jiang, X. Y. Zou, L. L. Yin, J. Cheng, Three-dimensional broadband acoustic illusion cloak for sound-hard boundaries of curved geometry. *Sci. Rep.* **6**, 36936 (2016).

16. F. Monticone, A. Alù, Do cloaked objects really scatter less? *Phys. Rev. X* **3**, 041005 (2014).

17. R. Fleury, F. Monticone, A. Alù, Invisibility and cloaking: Origins, present, and future perspectives. *Phys. Rev. Appl.* **4**, 037001 (2015).

18. W. Kan, M. Guo, Z. Shen, Broadband unidirectional invisibility for airborne sound. *Appl. Phys. Lett.* **112**, 203502 (2018).

19. D. A. B. Miller, On perfect cloaking. *Opt. Express* **14**, 12457–12466 (2006).

20. F. G. Vasquez, G. W. Milton, D. Onofrei, Broadband exterior cloaking. *Opt. Express* **17**, 14800–14805 (2009).

21. Q. Ma, Z. L. Mei, S. K. Zhu, T. Y. Jin, T. J. Cui, Experiments on active cloaking and illusion for laplace equation. *Phys. Rev. Lett.* **111**, 173901 (2013).

22. B. O. Zhu, K. Chen, N. Jia, L. Sun, J. Zhao, T. Jiang, Y. Feng, Dynamic control of electromagnetic wave propagation with the equivalent principle inspired tunable metasurface. *Sci. Rep.* **4**, 4971 (2015).

23. M. Selvanayagam, G. V. Eleftheriades, Experimental demonstration of active electromagnetic cloaking. *Phys. Rev. X* **3**, 041011 (2013).

24. P. Ang, G. V. Eleftheriades, Active cloaking of a non-uniform scatterer. *Sci. Rep.* **10**, 2021 (2020).

25. C. House, J. Cheer, S. Daley, An experimental investigation into active structural acoustic cloaking of a flexible cylinder. *Appl. Acoust.* **170**, 107436 (2020).

26. T. S. Becker, D.-J. van Manen, C. M. Donahue, C. Bärlocher, N. Börsing, F. Broggini, T. Haag, J. O. A. Robertsson, D. R. Schmidt, S. A. Greenhalgh, T. E. Blum, Immersive wave propagation experimentation: Physical Implementation and one-dimensional acoustic results. *Phys. Rev. X* **8**, 031011 (2018).

27. F. Broggini, M. Vasmel, J. O. A. Robertsson, D.-J. van Manen, Immersive boundary conditions: Theory, implementation, and examples. *Geophysics* **82**, T97–T110 (2017).

28. D.-J. van Manen, J. O. A. Robertsson, A. Curtis, Exact wave field simulation for finite-volume scattering problems. *J. Acoust. Soc. Am.* **122**, EL115–EL121 (2007).

29. D.-J. van Manen, M. Vasmel, S. Greenhalgh, J. O. A. Robertsson, Broadband cloaking and holography with exact boundary conditions. *J. Acoust. Soc. Am.* **137**, EL415–EL421 (2015).

30. Y. Wang, Frequencies of the Ricker wavelet. *Geophysics* **80**, A31–A37 (2015).

31. T. S. Becker, "Immersive wave experimentation: Linking physical laboratories and virtual simulations in real-time," thesis, ETH-Zürich, Switzerland (2020).

32. M. Redwood, *Mechanical Waveguides: The Propagation of Acoustic and Ultrasonic Waves in Fluids and Solids with Boundaries* (Pergamon Press, 1960).

33. T. S. Becker, N. Börsing, T. Haag, C. Bärlocher, C. M. Donahue, A. Curtis, J. O. A. Robertsson, D.-J. van Manen, Real-time immersion of physical experiments in virtual wave-physics domains. *Phys. Rev. Appl.* **13**, 064061 (2020).

34. M. Vasmel, J. O. A. Robertsson, D.-J. van Manen, A. Curtis, Immersive experimentation in a wave propagation laboratory. *J. Acoust. Soc. Am.* **134**, EL492–EL498 (2013).

35. H. R. Thomsen, M. Molerón, T. Haag, D.-J. van Manen, J. O. A. Robertsson, Elastic immersive wave experimentation: Theory and physical implementation. *Phys. Rev. Res.* **1**, 033203 (2019).

36. N. Börsing, T. S. Becker, A. Curtis, D.-J. van Manen, T. Haag, J. O. Robertsson, Cloaking and holography experiments using immersive boundary conditions. *Phys. Rev. Appl.* **12**, 024011 (2019).

37. X. Li, T. Becker, M. Ravasi, J. Robertsson, D.-J. van Manen, Closed-aperture unbounded acoustics experimentation using multidimensional deconvolution. *J. Acoust. Soc. Am.* **149**, 1813–1828 (2021).

38. X. Li, J. Robertsson, A. Curtis, D.-J. van Manen, Compensating for source directivity in immersive wave experimentation. *J. Acoust. Soc. Am.* **146**, 3141–3158 (2019).

39. C. E. Shannon, Communication in the presence of noise. *Proc. IRE* **37**, 10–21 (1949).
